# Developmental co‐occurrence of psychopathology dimensions in childhood

**DOI:** 10.1002/jcv2.12100

**Published:** 2022-09-05

**Authors:** Andrea G. Allegrini, Toos van Beijsterveldt, Dorret I. Boomsma, Kaili Rimfeld, Jean‐Baptiste Pingault, Robert Plomin, Meike Bartels, Michel G. Nivard

**Affiliations:** ^1^ Social, Genetic and Developmental Psychiatry Centre Institute of Psychiatry Psychology and Neuroscience King's College London London UK; ^2^ Department of Clinical Educational and Health Psychology Division of Psychology and Language Sciences University College London London UK; ^3^ Department of Biological Psychology Faculty of Behavioral and Movement Sciences Vrije Universiteit Amsterdam Amsterdam THE NetherLand Eroupe; ^4^ Amsterdam Public Health (APH) Research Institute Amsterdam University Medical Centre Amsterdam THE NetherLand Eroupe; ^5^ Amsterdam Reproduction and Development (AR&D) Research Institute Amsterdam University Medical Centre Amsterdam THE NetherLand Eroupe

**Keywords:** comorbidity, development, genetic and environmental effects, psychopathology, sibling effects

## Abstract

**Background:**

Comorbidity between psychopathologies may be attributed to genetic and environmental differences between people as well as causal processes within individuals, where one pathology increases risk for another. Disentangling between‐person (co)variance from within‐person processes of psychopathology dimensions across childhood may shed light on developmental causes of comorbid mental health problems. Here, we aim to determine whether and to what extent directional relationships between psychopathology dimensions within‐person, and between individuals within families, play a role in comorbidity.

**Methods:**

We conducted random intercepts cross‐lagged panel model (RI‐CLPM) analyses to unravel the longitudinal co‐occurrence of child psychopathology dimensions, jointly estimating between‐person and within‐person processes from childhood to early adolescence (age 7–12). We further developed an extension of the model to estimate sibling effects within‐family (wf‐RI‐CLPM). Analyses were separately conducted in two large population‐based cohorts, TEDS and NTR, including parent‐rated measures of child problem behaviours based on the SDQ and CBCL scales respectively.

**Results:**

We found evidence for strong between‐person effects underlying the positive intercorrelation between problem behaviours across time. Beyond these time‐varying within‐person processes accounted for an increasing amount of trait variance, within‐ and cross‐trait, overtime in both cohorts. Lastly, by accommodating family level data, we found evidence for reciprocal directional influences within sib‐pairs longitudinally.

**Conclusions:**

Our results indicate that within‐person processes partly explain the co‐occurrence of psychopathology dimensions across childhood, and within sib‐pairs. Analyses provided substantive results on developmental processes underlying comorbidity in behavioural problems. Future studies should consider different developmental timeframes to shed more light on the processes contributing to developmental comorbidity.

## Graphical abstract

Comorbidity between psychopathologies may be attributed to genetic and environmental differences between people as well as causal processes within individuals, where one pathology increases risk for another. Here, we aimed to determine whether and to what extent directional relationships between psychopathology dimensions within‐person, and within families, play a role in multivariate comorbidity. Our results indicate that within‐person, and to a lesser extent within‐family, processes partly explain the co‐occurrence of psychopathology dimensions in childhood, suggesting that both levels of analyses should be taken into account in developmental models of comorbidity.

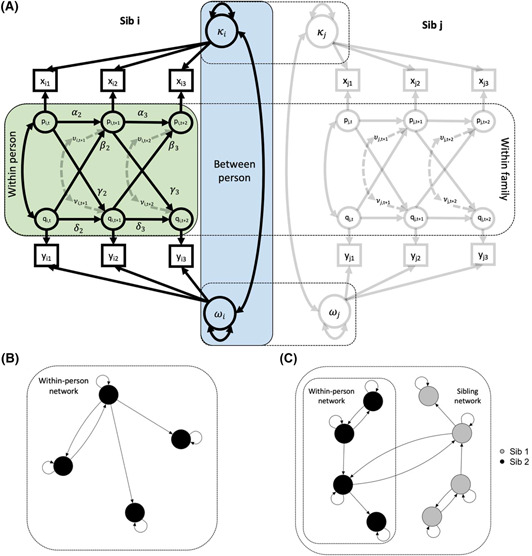

**Key points**

- Comorbidities could be the consequence of shared genetic and environmental influences, often conceptualized as individual differences between people, alternatively comorbidities could arise as a consequence of direct causal relations between traits.
- We sought to understand whether and to what extent directional relationships between psychopathology dimensions within‐person, and within sibling pairs, play a role in multivariate comorbidity.
- We investigated longitudinal data on measures of common psychopathologies from childhood to early adolescence (age 7–12), jointly estimating between‐person and within‐person processes across time.
- We found evidence for strong between‐person effects underlying the covariation between problem behaviours across development. On top of these, within‐person processes accounted for an increasing amount of trait variance overtime, with modest, but detectable, contributions of sibling effects.
- Our results indicate that within‐person, and to a lesser extent within‐family, processes partly explain the co‐occurrence of psychopathology dimensions in childhood.

## INTRODUCTION

The onset of many psychiatric disorders can be traced back to childhood or adolescence (Akingbuwa et al., 2020; Jansen et al., 2018; Kessler et al., 2005; Riglin et al., 2019) and 50% of individuals with a mental health diagnosis are likely to be diagnosed with one or multiple other disorders within a year time (Kessler et al., 1994, 2005). Probing the nature of co‐morbidity as a population phenomenon in childhood, and within individuals across development, is critical for our understanding of the development of psychopathology.

Common predispositions, such as genetic and environmental factors shared across traits (Allegrini et al., 2019; Caspi et al., 2014; Grotzinger et al., 2019; Pettersson et al., 2013), and random developmental differences (Molenaar et al., 1993) exist in competition to explain individual differences in developmental comorbidity. These influences in tandem can contribute to the ubiquitous covariation between psychopathology traits over the life course (Caspi et al., 2020). Beyond these influences, however, within person processes might be at play, such as direct, causal, relationships between psychopathology dimensions that can precipitate later development of other psychopathologies. That is the observed correlation structure between psychopathology traits could (partly) arise from an underlying network of causal effects (Borsboom, 2017).

It remains unclear to what extent the correlation between psychopathologies is the product of correlated fundamental differences between individuals on stable traits (stable across the life course), or the product of a causal process within individuals where the temporal state of one variable (e.g. mood) causally influences the state of another variable (e.g. attention), inducing the observed correlation. These two processes are not mutually exclusive and it is conceivable that both might play a role in the development of psychopathological comorbidity.

Disentangling between‐person from within‐person processes of psychopathology traits across childhood can shed light on putative causes of comorbidity, yielding insights into developmental pathways underlying mental health problems. By separating between and within person processes, we can tap into temporal directed effects between psychopathologies, adjusted for time invariant overarching (confounding) factors that can lead to spurious or biased (cross)lagged effect estimates (see Hamaker et al., 2015; Hamaker et al., 2020). These two levels of analyses are typically conflated in cross‐sectional designs and in the traditional cross lagged panel model (CLPM) (Hamaker et al., 2015). In turn, by investigating multi‐trait within‐person dynamics of psychopathology across development we can gain insights into mechanisms underlying developmental comorbidity (Hamaker et al., 2020), for example, uncovering mediators linking (within‐person) fluctuations in one psychopathology to another overtime.

Here we set out to investigate the longitudinal, directional associations between psychopathology dimensions from childhood to early adolescence (ages 7–12). We employ the random intercept cross‐lagged panel model (RI‐CLPM), to formally model within‐person processes across time (Hamaker et al., 2015), accounting for stable between‐person differences, such as genetic and environmental effects, that are not of a time‐varying nature.

Previous work attempting to separate within‐ and between‐level processes of psychopathology has focused on the bidirectional relationship between externalizing and internalizing behavior (Murray et al., 2020; Oh et al., 2020), externalizing and internalizing together with IQ (Flouri et al., 2019), anxiety and ADHD (Murray et al., 2022), and more recently between internalizing, externalizing, and attention problems (Richards et al., 2022). Here, we investigate reciprocal relationships between multiple psychopathology dimensions including externalizing‐, attention‐, internalizing‐, and social‐problems, across two different questionnaires (the Strengths and Difficulty Questionnaire and the Child Behaviour Checklist; Achenbach et al., 2017, Goodman, 1997) in two large population‐based cohorts (the Twin Early Development Study and the Netherlands Twin Register). Previous research typically aimed at disentangling bidirectional relationships between psychopathologies (e.g. internalizing and externalizing), often within diverse research designs. For example, considering different developmental timeframes, time‐lags (e.g. narrower), informants (e.g. teacher raters), or models to separate within‐ and between‐person effects. The pre‐registered aim of the present study (see https://osf.io/dtvc8/) is exploratory, as we do not assume the observed relations reflect causal relationships between measures of psychopathology. Rather we aim to determine whether and to what extent directional relationships between multiple measures of psychopathology jointly underly developmental comorbidity. The presence of within‐person effects between psychopathologies over time is viewed as a consequence of direct causal effects between these features of psychopathologies, but insufficient evidence to conclude those relations are in fact causal.

In supplementary analyses we further extend our investigation of developmental comorbidity to the family level. Given that individuals, and their psychopathologies, do not develop in a vacuum, but rather are nested in social structures (often, but not always, primarily in a nuclear family), we can extend the contrast of between‐person individual differences and within‐person processes to the family. Members of a family, siblings in particular, are known to resemble each other owning to shared environmental factors, like similarities in their upbringing, the means their parents had available to support their development, their cultural capital, and shared heritable influences. Similarities between siblings in terms of their symptoms of psychopathology and co‐morbidities can be attributed to heritable influences or influences of the shared environment. However, there are likely direct interactions between siblings, where age specific symptoms in one sibling could precipitate mental health symptoms in the other sibling at a later time (see Supporting Information). These interactions have family wide implications, where psychopathology networks within an individual can have an effect on the network of psychopathologies of another family member, such as their siblings, with cascading effects across development. We therefore extend the RI‐CLPM to accommodate similarities between siblings, while concurrently allowing for siblings directly influencing each other's problem behaviours. We test whether similarities in symptoms of psychopathology between siblings overtime are a function of correlated stable traits, due to heritable or environmental factors, or direct mutual influences of the behaviors of one sibling on the other.

## METHODS

### Samples

We analysed data from the Twin Early Development Study (TEDS (Rimfeld et al., 2019), a large longitudinal population‐based study involving 16,810 pairs of twins born in England and Wales between 1994 and 1996. Here we focused on parent rated (mainly maternal) psychopathology measures administered when the twins were aged 7, 9, and 12 (*n* = 3385–7758 twins depending on measure, Table S1a). We then conducted the same set of analyses in the Netherlands Twin Register (NTR; Ligthart et al., 2019), a longitudinal population‐based sample with new twins data added every year. The Young‐NTR (Bartels et al., 2007) contains data on twins from birth onwards. Data collection is driven by birth cohort. Here we also focused on maternal ratings when the twins were aged 7, 10, and 12 (*n* = 8738–12,710 twins, Table S1b).

### Measures

In TEDS we employed the parent‐rated version of the Strength and Difficulties Questionnaire (SDQ; Goodman, 1997) comprising four scales indexing emotional symptoms (anxiety and depression), conduct problems, hyperactivity/inattention, and peer relationship problems. In NTR we applied the same analysis pipeline using the mother‐rated Child Behaviour Checklist (CBCL; Achenbach et al., 2017) at similar age intervals as in TEDS (mean age 7, 9, and 12, vs. mean age 7, 10 and 12). The CBCL comprises eight syndrome scales including aggressive behaviour, anxious/depressed, attention problems, rule‐breaking behaviour, somatic complaints, social problems, thought problems, and withdrawn/depressed. For consistency we use approximately equivalent scales between the SDQ (available in TEDS) and the CBCL (available in NTR), that is, conduct problems, hyperactivity/inattention, emotional problems, and peer problems in SDQ, and externalizing, inattention, internalizing, and social problems in CBCL. These are henceforth respectively called externalizing (EXT), attention (ATT), internalizing (INT), and social (SOC) problems for both TEDS and NTR, with subscript 1, 2, or 3 to indicate the different measurement occasions (age 7, 9/10, and 12 respectively). SDQ and CBCL scales have been shown to correlate highly with each other (*r* = 0.59 to 0.84) in childhood (Goodman & Scott, 1999) and to be highly comparable across cohorts. Scales from the SDQ and CBCL show similar patterns of co‐occurrence in TEDS and NTR with aggression (Bartels et al., 2018) and a similar genetic architecture for externalizing (Porsch et al., 2016).

To create non‐overlapping age bins across measurement occasions, we excluded individuals much older or younger than their corresponding age bin. In practice we limited the age range of each bin to obtain approximately equal intervals with non‐overlapping observations between lags: mean age 7.06 [range 5.57–7.98], 9.01 [8.08–9.96], and 11.35 [10–13.5] for TEDS; and mean age 7.45 [6.2–9]; 9.98 [9–11] and 12.26 [11–13.5] for NTR. Then, we derived z‐standardised residuals for each SDQ and CBCL subscale regressed on sex and age (at each age bin). We then analysed standardized residuals in structural equation models. Table S1 report descriptive statistics of all measures used in the study by cohort.

### Analyses

#### RI‐CLPM: Estimating within‐person effects

**FIGURE 1:**
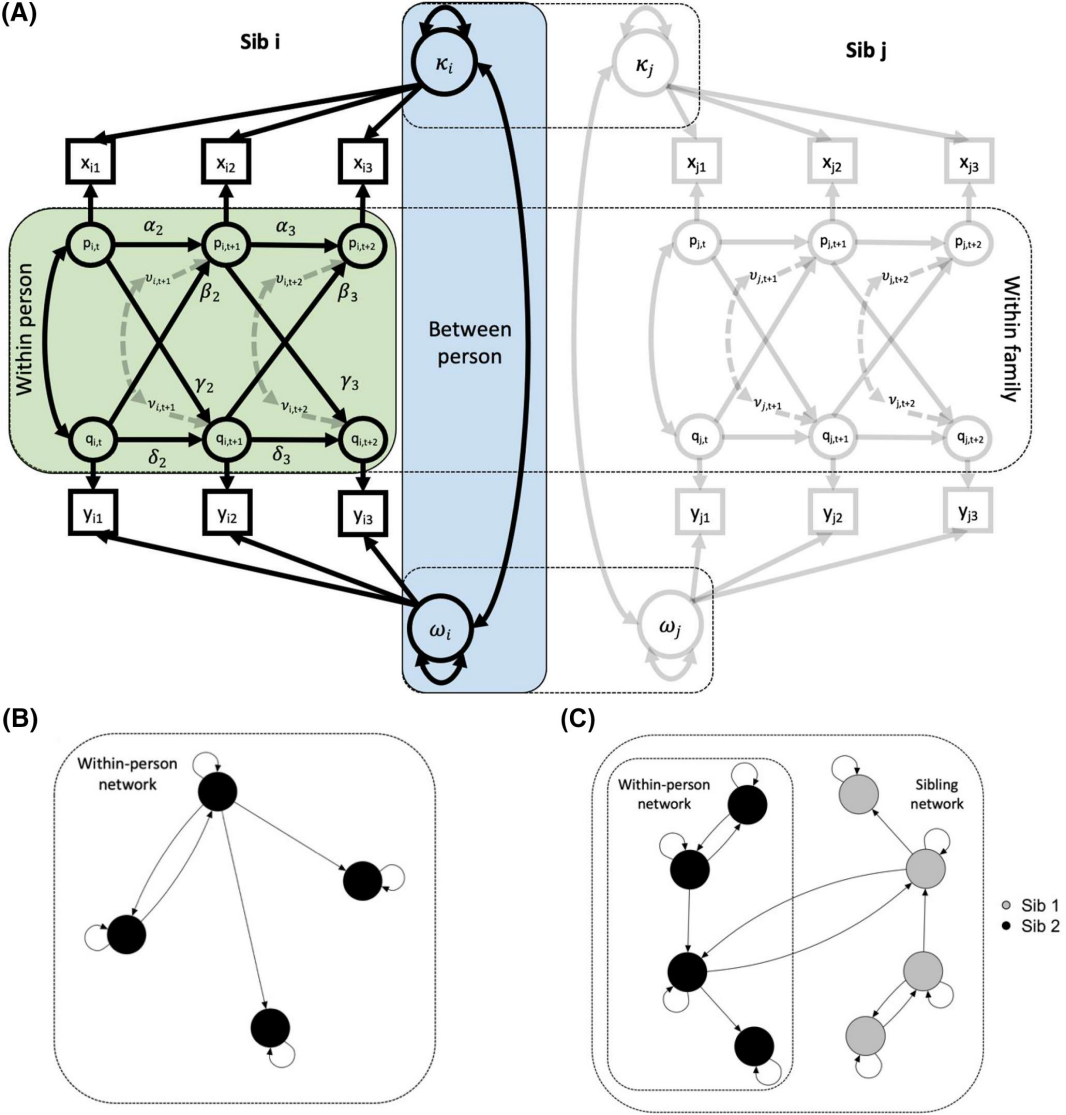
wf‐RI‐CLPM. Panel A: Schematic depiction of the random‐intercept cross‐lagged panel model extended to sibling pairs (i.e. within‐family; wf‐RI‐CLPM) for two traits and three measurements. The green and blue shades respectively outline within‐person and between‐person effects (for sibling ‘i’) modelled in the RI‐CLPM. The dashed contours outline between‐sibling (i and j) effects (regressions) and covariances (between residuals, and between random intercepts) modelled in the wf‐RI‐CLPM. Note: covariances and regression paths between siblings not shown for clarity. Actual models tested included 4 traits and 3 measurement occasions for both the RI‐CLPM and the wf‐RI‐CLPM. Panel B: Example of within‐person network depicting lagged relationships across four measurements. Nodes represent time deviations (e.g. p and q in panel A) and edges represent directional (temporal) effects within‐trait (self‐pointing arrows; αs and δs in panel A) or cross‐trait (βs and γs in panel A). Panel C: Example of sibling network where lagged relationships are allowed form one person to another, connecting within person networks. Network plots were created with the r package qgraph (Epskamp et al., 2012)

We modelled psychopathology measures longitudinally fitting a RI‐CLPM (Hamaker et al., 2015; Mund & Nestler, 2019) to test for the presence of within‐person directional influences of psychopathological traits over time. The RI‐CLPM (Figure 1A) is an alternative to the widely used CLPM. A detailed critique of the CLPM is available elsewhere (Hamaker et al., 2015). Briefly, the CLPM cannot disentangle within‐person effects over time from between‐person stable effects. By including random intercepts, the RI‐CLPM allows to separate within and between individual effects at each measurement. There are several generalizations and alternatives to the RI‐CLPM described elsewhere (Epskamp, 2020; Mund & Nestler, 2019; Zyphur et al., 2019).

Figure 1 includes a schematic representation of a RI‐CLPM for two traits and three measurements. The random intercepts (ω or κ) represent the stable between‐person trait like influences across time, while the fixed intercepts estimate the group mean level at each measurement for a trait. The individual level deviation at each measurement is estimated by the latent variables *p*_*it*_ and *q*_*it*_, while α_2_ and α_3_ (and δ_2,_ δ_3_) estimate the within person carry‐over effects between measurement x_it_ and measurement x_it+1_. Finally, the cross‐lagged paths, β_2_ and β_3_ (and γ_2_, γ_3_) estimate the effects of the within‐person deviation over the expected score (given by the temporal means μ_*t*_ or π_*t*_ and the random intercepts ω or κ) for a trait at one measurement, on the deviation from the expected score for another trait at another measurement. These latter effects are reciprocal directed influences between traits from one time point to another, adjusted for group‐level mean differences, between‐person trait‐level stability over time, and within‐person carry‐over effects. In other words, any effect found in this regard is indicative of processes acting at the individual level, indexing the extent to which a person's unusually high (or low) levels for a trait at one time point are predictive of the person's unusually high (or low) levels for another trait later in time (Hamaker et al., 2015).

We present results for TEDS followed by a replication of analyses in NTR: for both TEDS and NTR we conducted the same procedures as described below. First, we fit a model in which we constrain variances and regressions to be the same across time and, based on standard model fit indices (i.e. χ^2^ test, AIC, BIC, RMSEA, SRMR, CFI) and compare it to an unconstrained model in which we let these parameters vary freely overtime. While a constrained model has easier interpretation, an unconstrained model may be warranted to better capture developmental processes, as our observations are not taken at close intervals (time‐lags are ∼2 years).

The best fitting model is then carried forward in the analyses. Within this model we specifically test whether the longitudinal (cross‐lagged) paths (β_2_, β_3_ and γ_2_, γ_3_), in the example diagram in Figure 1, are significantly different from 0. We remove paths for which we do not find evidence against the null‐hypothesis of no effect (using the Benjamini‐Hocberg FDR procedure for multiple testing; Cribbie, 2007), and refit this ‘pruned’ model. Finally, we fit a ‘null’ model with all (cross‐lagged) longitudinal paths removed. We then evaluate the difference in model fit between these nested models. To study sex specific developmental processes, we perform multi‐group analyses by sex in the full models (i.e. not pruned for non‐significant paths), using parameter constraints to test whether regressions differ between males and females. All RI‐CLPM analyses were conducted in unrelated individuals, selecting one random sibling per twin pair.

#### Within family RI‐CLPM: Estimating reciprocal sibling effects

In supplementary analyses, we extended the RI‐CLPM to family data, by considering twin pairs instead of restricting analyses to unrelated individuals. Specifically, we extended the model to include monozygotic (MZ) and dizygotic (DZ) twin pairs, employing different model specifications depending on zygosity. We call this model within‐family RI‐CLPM (wf‐RI‐CLPM). This extension aims to further separate reciprocal directional influences between siblings (within families) from similarities between siblings that arise through shared (genetic or environmental) influences that exist in a family. Furthermore, by estimation of twin correlations, inferences can be made on the genetic and environmental contributions at the level of time‐invariant overarching stable traits, and age specific residuals. In practice here we take the concept of a network model from an individual to a family level, while controlling for the fact that family members are related to each other.

First, we run a multi‐group RI‐CLPM on MZ and DZ twins. We fix random intercepts, variances, covariances, and regressions within individuals to be equal across zygosity and for twin 1 and twin 2, while we let between‐person covariances of random intercepts and age specific residuals vary between zygosity groups (MZ vs. DZ). Finally, between‐sibling regressions (Figure 1C) are constrained to be equal across zygosity.

Similar to the RI‐CLPM, the measurement model can be expressed as follows for sibling 1 (i) and sibling 2 (j):

(1) $$x_{it}=\mu_{t}+k_{i}+\;p_{it}$$

$$\;y_{it}=\pi_{t}+\omega_{i}+\;q_{it}$$

$$x_{jt}=\mu_{t}+k_{j}+\;p_{jt}$$

$$\;y_{jt}=\pi_{t}+\omega_{j}+\;q_{jt}$$

Where μandπ are the group means at measurement *t* for trait *x*_*t*_ and trait *y*_*t*_, κ and ω are the between‐person latent factors (random intercepts) for the two traits respectively measured over time. *p*_*t*_ and *q*_*t*_ are the deviations from a person expected score (i.e. κ+μ). In the wf‐RI‐CLPM the within person variance for the random intercepts is the same for both MZ and DZ, and twin 1 (*i*), twin 2 (*j*):

(2) $$\text{var}\left(\omega_{i}\right)\;=\;\text{var}\left(\omega_{j}\right)\;\text{and}\;\text{var}\left(k_{i}\right)=\;\text{var}\left(k_{j}\right)$$

While the within‐ and cross‐trait covariance between two members of a twin pair is freely estimated across zygosity groups. Expanding on the equation for change of the RI‐CLPM (Hamaker et al., 2015) the model for the longitudinal deviations can be expressed as follows for a given MZ or DZ pair (example for two traits *p* and *q*):

Trait *p*:

(3) $$p_{it\;=}\alpha_{t}p_{i,t-1}+\beta_{t}q_{i,t-1}+\alpha_{t}p_{ji,t-1}+\beta_{t}q_{ji,t-1}+v_{it}$$

$$p_{jt\;=}\alpha_{t}p_{j,t-1}+\beta_{t}q_{j,t-1}+\alpha_{t}p_{ij,t-1}+\beta_{t}q_{ij,t-1}+v_{jt}$$

Trait q:

(4) $$q_{it\;=}\delta_{t}q_{i,t-1}+\gamma_{t}p_{i,t-1}+\delta_{t}q_{ji,t-1}+\gamma_{t}p_{ji,t-1}+\upsilon_{it}$$

$$q_{jt\;=}\delta_{t}q_{j,t-1}+\gamma_{t}p_{j,t-1}+\delta_{t}q_{ij,t-1}+\gamma_{t}p_{ij,t-1}+u_{jt}$$

The part of the equation *p*_*it =*_ α_*t*_ *p*_*i,t*−1_ + β_*t*_ *q*_*it*−1_ + υ_*it*_ is akin to the equation for the time deviations of RI‐CLPM in (Hamaker et al., 2015). Where α_*t*_ *p*_*i,t*−1_ and β_*t*_ *q*_*i,t*−1_ are the within‐person regressions within‐trait and cross‐trait (respectively) for trait *p* in twin 1 (subscript i), while δ_*t*_ *q*_*j,t*−1_ and γ_*t*_ *p*_*j,t*−1_ are the within‐person regressions within‐trait and cross‐trait (respectively) for trait *q* in twin 2 (subscript j). υ_*it*_ is the residual trait variation for trait *p* in twin 1 and ν_jt_ is the residual trait variance of trait *q* in twin 2. The second part of the equation differs in the following ways: α_*t*_ *p*_*ji,t*−1_ and β_*t*_ *q*_*ji,t*−1_ are the between‐sibling regressions within‐trait and cross‐trait (respectively) of twin 1 on twin 2 (subscript ji), for trait *p*. While δ_*t*_ *q*_*ij,t*−1_ and γ_*t*_ *p*_*ij,t*−1_ are the between‐sibling regressions within‐trait and cross‐trait (respectively) of twin 2 on twin 1 (subscript ij), for trait *q*. Note that in the model specification α_t_ *p*_*ji,t*−1_ = α_*t*_ *p*_*ij,t*−1_ and γ_t_p_*jit*−1_ = γ_*t*_ *p*_*ij,t*−1_ (similarly δ_*t*_ *q*_*ji,t*−1_ = δ_*t*_ *q*_*ij,t*−1_ and β_*t*_ *q*_*jit*−1_ = β_*t*_ *q*_*ij*,*t*−1_) within and across zygosity. In this context α_t_ and δ_t_ represents the between sibling effects within‐trait and γ_t_ and β_t_ index between sibling effects cross‐trait, after accounting for within‐person changes from one time point to the next, group‐mean level at each time point, and between‐person differences overtime. Here the main interest is in both within‐trait cross‐twin and between‐trait cross‐twin effects (conversely in the standard RI‐CLPM the main interest is in within‐person cross‐lagged effects). Figure 1 is a schematic depiction of the wf‐RI‐CLPM for two traits and three measurements in a sib pair.

As a result of fitting the wf‐RI‐CLPM to twin data, we can compare MZ and DZ twin correlations to estimate the relative contributions of genetic and environmental components to stable between‐person differences (indexed by the random intercepts) as well as the age specific within person residual effects (see Supporting Information).

For the wf‐RI‐CLPM we take a three‐step procedure akin to the RI‐CLPM analyses in unrelated individuals. First, we fit a full (unconstrained) model estimating all within‐person within and cross‐trait effects, and all between‐sibling within and cross‐trait effects. We then formally compare this model with two nested models: (1) a pruned model in which we drop all non‐significant between‐sibling paths (using a nominal significance threshold of α= 0.05); and (2) a ‘null’ model in which we drop all between‐sibling paths. We use the “qgraph” R package (Epskamp et al., 2012) to plot networks of relationships between random intercepts (between‐person network), and regression estimates within‐person (within‐person network; Figure 1B) and between siblings (sibling network; Figure 1C).

For all analyses we used a Maximum Likelihood estimator with robust standard errors (MLR), and full information maximum likelihood to treat missing data (FIML). Analyses were performed in Rstudio (v1.2.1335), structural models were specified using Lavaan (v0.6–5). See https://osf.io/dtvc8/ for the preregistered protocol. We provide code and summary level data (observed variance/covariance matrices) to replicate results from the RI‐CLPM and wf‐RI‐CLPM models, as well as a R function to automatically create wf‐RI‐CLPM lavaan models for any number of traits and measurement occasions: https://github.com/AndreAllegrini/wfRI‐CLPM/.

## RESULTS

Tables S2 show bivariate correlations between all study variables. Both the full constrained and unconstrained models showed an excellent fit (Table S3). In TEDS the chi‐square test favoured the constrained over the unconstrained model: Δ*χ* ^2^ (26) = 32.688, *p* = 0.170. However, CFI and RMSEA favoured the unconstrained model. Upon inspection of cross‐lagged regressions it was evident that the pattern of relationships differed between the two time‐lags (age 7‐9 and age 9–12; see below) indicating a developmental change in the within‐person process overtime. As such we carried forward the unconstrained model: χ^2^ (6) = 10.550, *p* = 0.103, RMSEA = 0.009, SRMR = 0.004, CFI = 0.999. In NTR the full unconstrained model had an excellent fit too and was favoured over the constrained model based on the chi‐square test Δ*χ* ^2^ (26) = 64.238, *p* = 4.403E‐5 and standard fit indices (Table S3c): χ^2^ (6) = 54.971, *p* = 2.2E‐16, RMSEA = 0.022, SRMR = 0.00, CFI = 0.999.

### Between‐person stable effects

**FIGURE 2:**
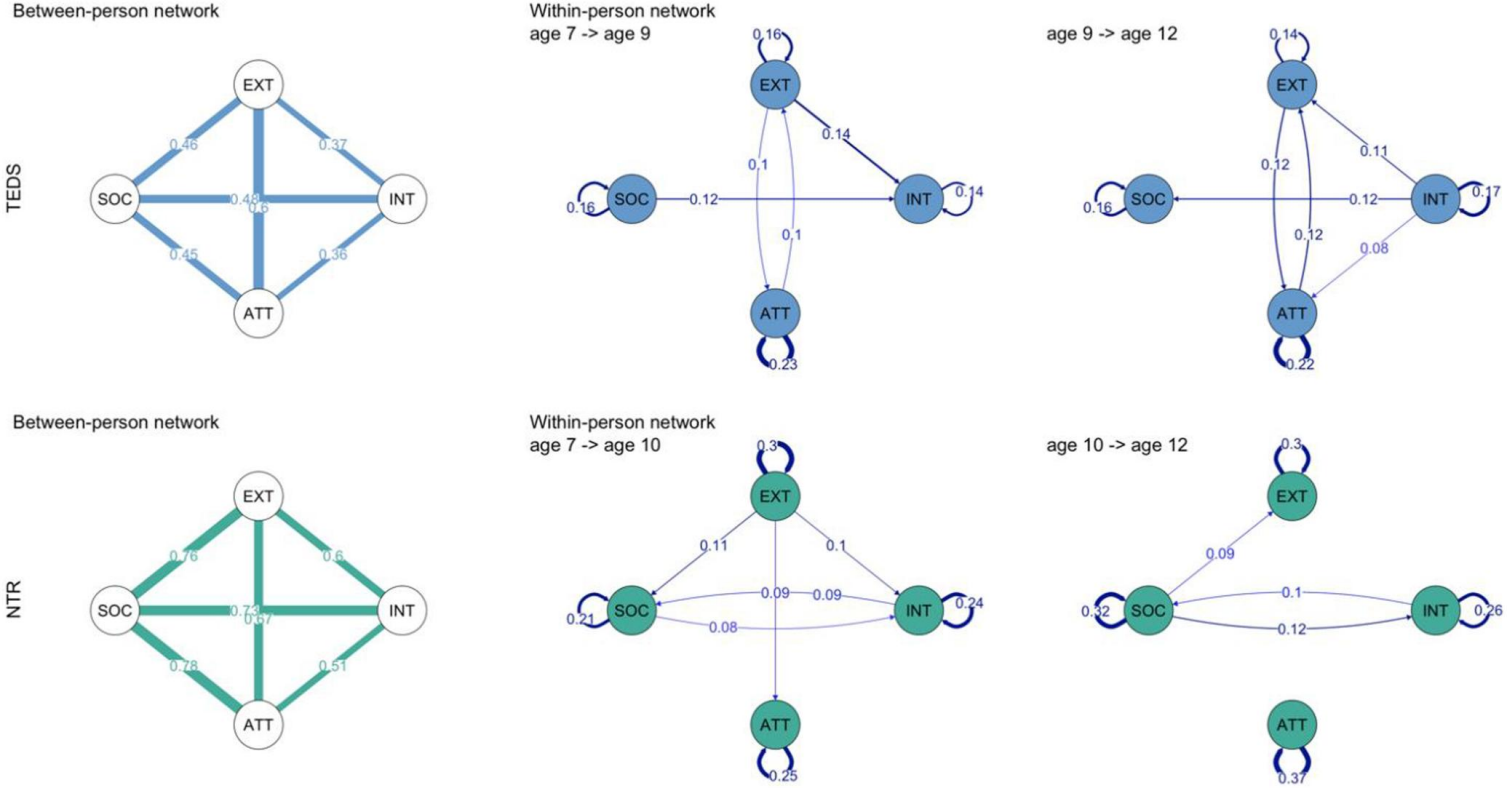
Between‐person and within‐person (directed) networks of relationships in TEDS (blue) and NTR (green) obtained from the RI‐CLPM. Nodes represent the measure of interest (the random intercept in the case of between‐person networks, and residual deviation of the measurement occasion for the within‐person network). Edges width and labels indicate and quantify the strength of relationships between nodes, and in the case of within‐person networks also the temporal direction of the effect. For every time lag (7−>9 and 9−>12 for TEDS; 7–10 and 10−>12 for NTR) edges represent directional effects within‐trait (self‐pointing arrows; αs and δs in Schematic figure 1A) or cross‐trait (βs and γs in Schematic Figure 1A). SDQ/CBCL acronyms: ATT, hyperactivity‐inattention/inattention; INT, emotional problems/internalizing; EXT, conduct/externalizing; SOC, peer problems/social problems. All edges shown survived FDR correction for multiple testing. Network plots were created with the r package qgraph (Epskamp et al., 2012)

In TEDS, between person individual differences as indexed by the random intercepts accounted for a substantial proportion other variation of the constructs under study (EXT = 48%, ATT = 56%, INT = 45%, SOC = 42%). There were positive correlations (ranging from 0.36 to 0.60, see between‐person network in Figure 2) between all traits indicating that higher rating for a particular child problem behaviour across time also tended to be higher for other problem behaviours across the three measurements waves. These between person correlations were twice the magnitude of within person correlations at any given time point (Table S4a, and Figure S1).

Consistent with TEDS findings, in NTR we observed substantial between‐person effects for all traits (EXT = 57%, ATT = 54%, INT = 44%, SOC = 43%) and strong positive correlations (Figure 2). Again, these were more than twice in extent the within person simultaneous correlations at any given time (Table S4b and Figure S1). We then considered evidence for within‐person effects.

### Within‐person time‐varying effects

We detected several positive directed effects between problem behaviours across time surviving FDR correction. These indicated the extent to which deviations from a person expected score in one problem behaviour at one time point (e.g. age 7) predicted deviations in the person's other problem behaviour at a subsequent time point (e.g. age 9), after accounting for stable between‐person differences and time‐varying carry‐over effects. Tables S5 report coefficients for all regressions in TEDS and NTR, Figure 2 shows network plots of directed within‐person relationships.

Within‐person networks evidenced a reciprocal pattern of relationships between several dimensions over time. Of note, for example, were reciprocal effects of externalizing and attention problems at both time lags in TEDS and between internalizing and social problems at both time lags in NTR. In both cases directed effects were of similar magnitude (*β* ∼ 0.1) indicating a positive loop overtime, rather than causal predominance of one variable over the other across time (See Figure 2).

**FIGURE 3:**
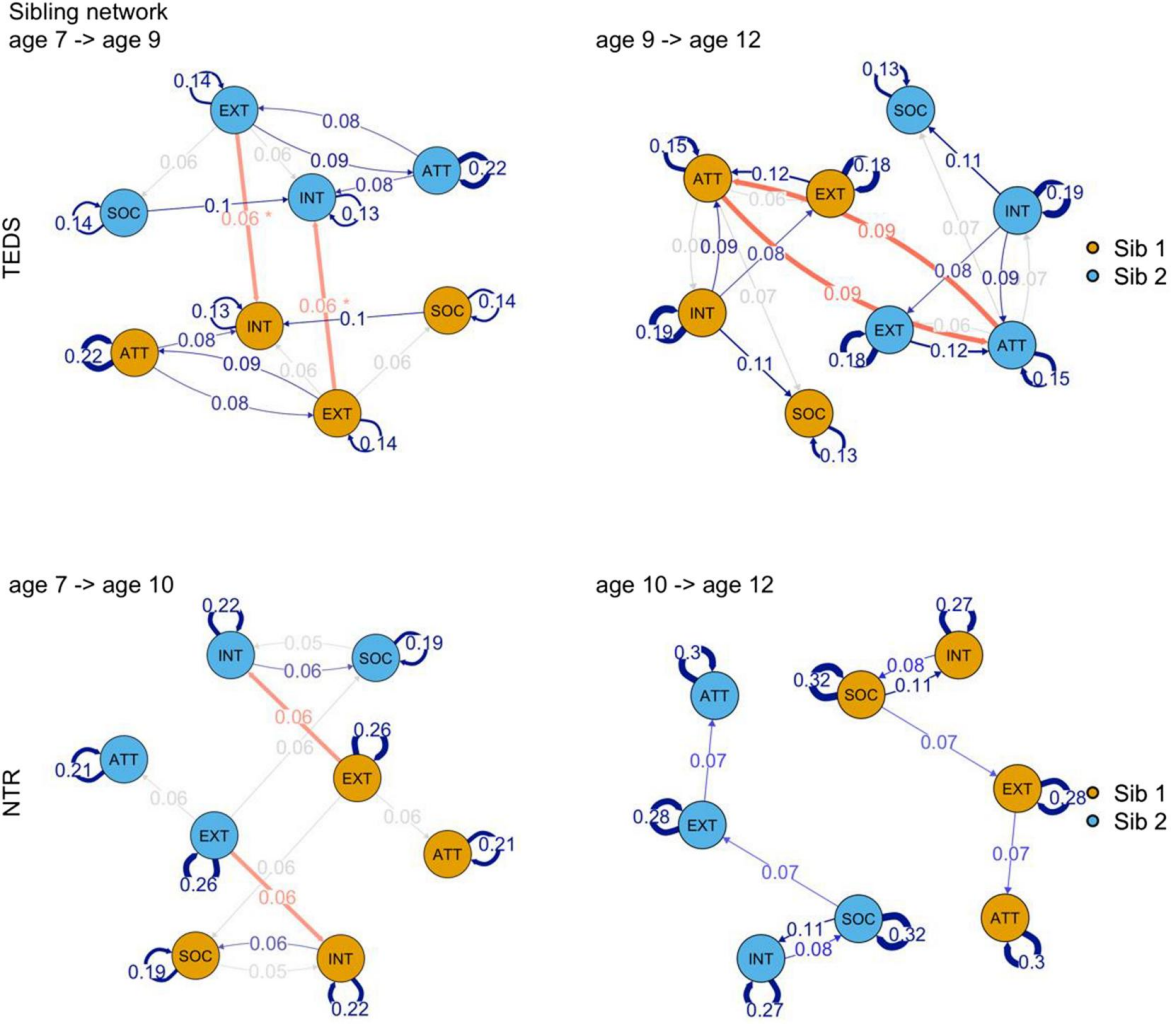
Sibling network plots in TEDS and NTR obtained from the wf‐RI‐CLPM. Figure shows within‐person networks for sibling pairs within‐families, with between‐sibling relationship represented by directed edges form one within‐person network to the other (highlighted in red for clarity). * = FDR correction for multiple testing; otherwise *p* < 0.05. For within person networks blue edges survived FDR correction for multiple testing, while gray edges correspond to nominal significance α< 0.05. SDQ/CBCL acronyms: ATT, hyperactivity‐inattention/inattention; EXT, conduct/externalizing; INT, emotional problems/internalizing; SOC, peer problems/social problems. Network plots were created with the r package qgraph (Epskamp et al., 2012)

**FIGURE 4:**
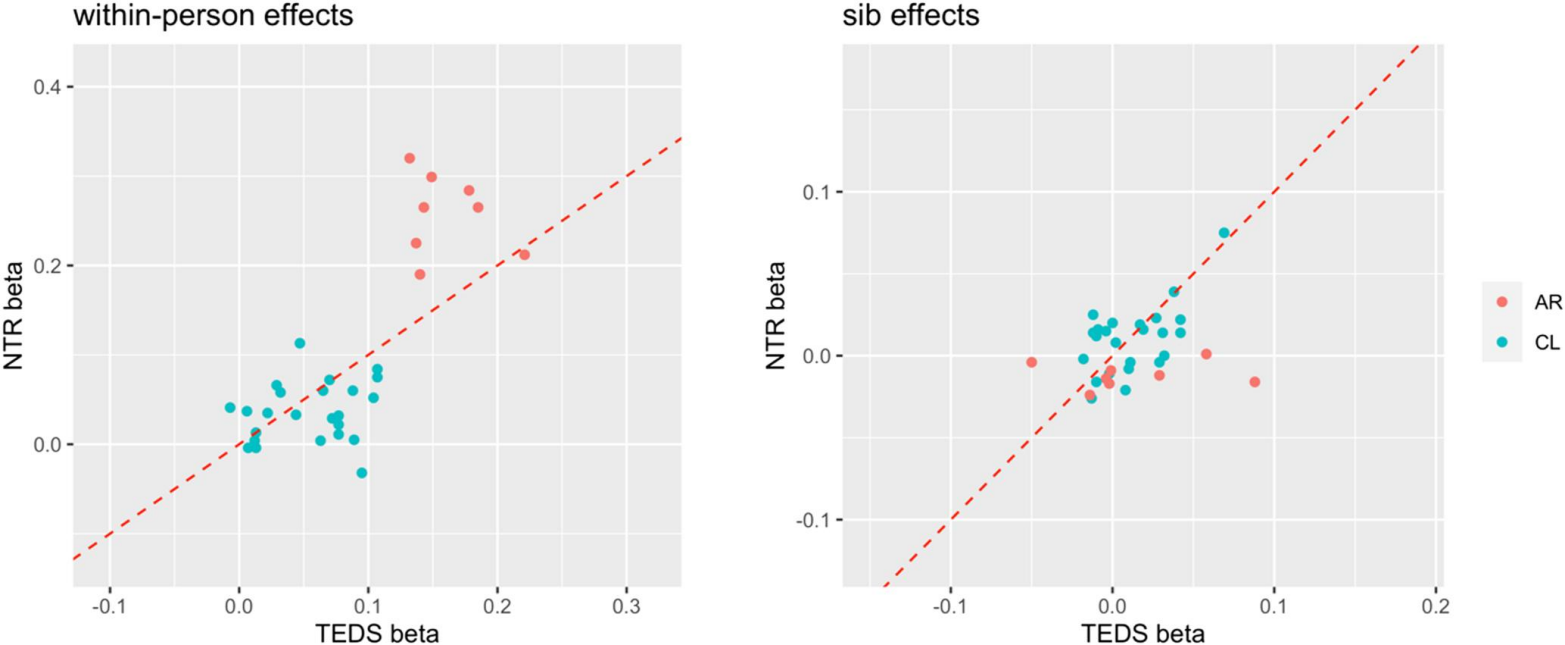
Comparison of effect sizes between NTR and TEDS. Left panel shows a comparison of within‐person effect sizes. Right panel shows effect size comparisons for sibling regressions. AR, autoregressive (carry‐over) effects; CL, cross‐lagged effects. Dashed line: *x* = y

The pattern of relationship emerging in the network plots partly overlapped between TEDS and NTR, specifically externalizing problems were predictive of internalizing (*β* = 0.140, se = 0.037, *p* = 4.49E‐5; and *β* = 0.100, se = 0.042, *p* = 5.39E‐3) and attention problems (*β* = 0.099, se = 0.034, *p* = 4.65E‐3; and *β* = 0.089, se = 0.037, *p* = 1.14E‐2) from age 7–9 (age 7–10 in NTR); while social problems predicted internalizing problems (*β* = 0.117, se = 0.034, *p* = 6.00E‐4; and *β* = 0.080, se = 0.032, *p* = 1.26E‐2) from age 7 to 9–10; in turn internalizing problems were predictive of social problems (*β* = 0.115, se = 0.040, *p* = 3.70E‐3; and *β* = 0.097, se = 0.033, *p* = 3.17E‐3) from age 9–10 to age 12. Figure 4 shows a comparison of within‐person effect sizes between TEDS and NTR, indicating the degree to which effects replicated across cohorts. Figure S2 shows the high concordance in effect size estimates between RI‐CLPM and wf‐RI‐CLPM.

Within person directed effects accounted for 4%–11% of the variance in TEDS and 10%–18% of the variance in NTR in the full models, depending on measure and wave (Table S6). Nested model comparisons with a pruned model in which only FDR adjusted parameters were included in the model (as depicted in Figure 2), and a null model in which all cross‐lagged paths were dropped, favoured the pruned model in TEDS and the full unconstrained model in NTR (Tables S3).

### Sex differences

While in TEDS we did not find evidence for sex differences as indicated by multigroup comparisons (Tables S7a–S7d), in NTR developmental differences between groups were evident (Tables S8a, S8b and Figure S3). On one hand, in males, within‐person within‐trait effects were stronger for internalizing and externalizing problems than in females. The network of cross‐lag relationship involved effects of externalizing on internalizing problems across time lags (age 7 to 10 and 10 to 12) and on attention problems from age 10 to 12. In turn internalizing and social problems were reciprocally predictive from age 10 to 12. On the other hand, in females we found only weak evidence (nominal significance) for within‐person effects, which suggested a role of externalizing problems only in the first time‐lag (on social and attention problems), with a more predominant role of social problems later in life (on externalinzing and inter problems; Figure S3).

### Sibling effects

Both in TEDS and NTR the full unconstrained models including sibling effects had an excellent fit (Table S9). Tables S10 show correlations for observed variables by zygosity. Figure S4 shows MZ versus DZ twin correlations for random intercepts and age specific residual variances for TEDS and NTR. Twin correlations were overall consistent with additive genetic variation. Falconer's equations were used to infer components of variance (see Supporting Information S2), either genetic or environmental. Figure S5 shows estimates derived by doubling the difference between MZ and DZ correlations. Overall, these were consistent with an additive model of genetic variance, and little shared environmental effects. For attention problems, genetic dominance effects were evident with the exception of age 9 and age 12 in TEDS. In TEDS for the ATT random intercept we found evidence of overdominance (*d* ^*2*^ effects exceeding 1) possibly due to a mix of true dominance effects and contrast effects (Supporting Information 1). In this regard, however, it should be noted that in previous work in NTR limited evidence was found for rater contrast effects after age 3 (Rietveld et al., 2003). Figure S5 depicts variance component estimates for latent stable traits, and age specific residual variances. The pattern of variance components estimates was not always consistent between TEDS and NTR. Inspection of the observed covariance matrix for DZs and MZs twins (Table S10) showed that the overdominance evident in TEDS was underlay by DZs correlations of ∼0 for ATT at age 7. On the other hand, the differing ADE versus ACE pattern for ATT at ages 9 and 12 for NTR versus TEDS might be attributable to differences in measures employed (see discussion).

Nested comparisons indicated that the pruned model, where only significant between‐sibling regression paths were retained, was to be favoured (Tables S9). Figure 3 shows siblings network plots where the concept of Figure 2 is extended to include regressions from one within‐sibling network to another, within a family (Tables S11).

In both TEDS and NTR we detected a positive cross‐trait between‐sibling effect at the first time lag of externalizing on internalizing problems (*β* = 0.063, se = 0.028, *p* = 1.30E‐2 for TEDS, and *β* = 0.064, se = 0.03, *p* = 1.24E‐2 for NTR). This indicated that higher externalizing problems for one sibling at age 7 longitudinally predicted higher internalizing problems for their sibling at a later time point (age 9 for TEDS and age 10 for NTR), after adjusting for within‐person time‐varying effects and between‐person individual differences, such as stable genetic or environmental confounds. We further detected a within‐trait between‐sibling effect of attention problems in TEDS (*β* = 0.087, se = 0.039, *p* = 2.32E‐2), this effect was however not evident in NTR (Tables S11 for all regression estimates, Table S12 for simultaneous correlations and residual variances).

## DISCUSSION

We investigated directional relationships between problem behaviours in two population‐based twin cohorts, separating within‐versus between‐person variation in a longitudinal design. We found that modelling within‐person cross‐trait relationships over time provides the best fit to the data, indicating that directed effects are an important source of covariation between problem behaviours in childhood after adjusting for stable between‐person effects.

The observation that psychopathology traits are positively intercorrelated across the lifespan (the so‐called positive manifold; Borg, 2018) has led some to conclude that a shared common cause, operationalized as a hierarchical factor model (labelled the p‐factor), is a parsimonious explanation for the co‐occurrence of psychopathologies (Caspi et al., 2014). However, observing the positive intercorrelation between psychopathologies isn't sufficient to establish its cause. In fact, it has been noted that it is a mathematical necessity that a hierarchical factor model fits the data well when observing a positive manifold (Van Bork et al., 2017). There is a variety of data generating processes that can give rise to the positive manifold between measures of psychopathology (Van Bork et al., 2017). For example, a compelling alternative for the p‐factor model is that proposed by the network approach of psychopathology, which poses that the observed correlation structure between psychopathology traits can arise from an underlying pattern of causal effects at the symptom level (Borsboom, 2017). The temporal causal relationships between symptoms would induce a positive correlation between psychopathology traits, which then appears consistent with the presence of a common cause.

Here, within a specific developmental timeframe, we provide evidence that the intercorrelation between psychopathology traits in childhood arise as a function of strong positively correlated between‐person processes, and many within‐person directional processes of modest effect. This suggests that both processes, common stable and time‐varying directed causal effects, might jointly shape developmental comorbidity in childhood. Importantly ‘small effects’ is not synonym of ‘inconsequential’, especially when multiple such effects concurrently accumulate over time (Götz et al., 2022). In fact, we found that within person processes, within‐ and cross‐trait, account for an increasing amount of trait‐variance over time, up to 11% for attention problems in TEDS, and up to 18% for attention problems and social problems in NTR. Developmentally, this is especially relevant under a network lens where activation of one node can have widespread cascading effects on the rest of the network over time.

Importantly, a central feature of the present study consisted in conducting analyses and replication across two large population‐based samples. Below we discuss robustness of within‐person developmental processes and consistency with previous evidence. First, in both cohorts we observed directed within‐person effects of externalizing problems at age 7 on internalizing problems at age 9–10. This finding parallels recent evidence in independent cohorts, investigating within person processes in the bivariate relationship between externalizing to internalizing problems across childhood (Murray et al., 2020; Oh et al., 2020). However, we also observe that from age 9–10 to 12 this relationship is reversed in TEDS, with higher internalizing problems linked to higher externalizing problems, and not subsistent in NTR, possibly owning to a change in developmental processes as children grow up.

Other related evidence provides mixed findings in this regard, with either bivariate relationships between externalizing and internalizing problems (Flouri et al., 2019), an inverse relationship between internalizing and externalizing in adolescence (Murray et al., 2020), or links between internalizing and externalizing emerging in early adulthood (Richards et al., 2022). These conflicting results might, partly, be a result of the developmental timeframe considered in the different studies. However, as noted earlier in the introduction and pointed out elsewhere (Richards et al., 2022), methodological differences between these studies make it challenging to draw conclusions in this regard. For example, previous studies mostly looked at bivariate relationships between psychopathologies, or concurrently estimated effects of different developmental traits, while here we jointly estimate effects between four different psychopathologies.

In both cohorts we found that social problems at age 7 were also predictive of internalizing at age 9–10. In turn, internalizing problems predicted social problems from age 9–10 to age 12 within‐person, indicating a positive feedback loop over time. Problematic relationships with peers in early childhood have been known to precipitate emotional problems later in childhood (Menting et al., 2015; Van lier & Koot, 2010). The present findings, in turn, suggest that targeting both internalizing symptoms and social problems early in childhood, could have an impact on their reciprocal relationship over time.

Finally, within‐person associations of externalizing on attention problems from age 7 to age 9–10 were evident in both cohorts, providing converging evidence with previous work employing a genetically sensitive design (Kuja‐halkola et al., 2015). This relationship was also evident in later adolescence in TEDS, consistent with work jointly analysing internalizing, externalizing and attention problems (Richards et al., 2022), from adolescence through early adulthood. It is of note, however, that this relationship disappeared in early adulthood in this previous recount.

An aim for follow‐up studies will be to explore different timeframes, not only in terms of developmental periods, but also in terms of time‐lags considered: weeks versus months versus years. This investigation should also be coupled with the use of different informants within the same study, as well as type of assessments, when jointly considering a number of different psychopathologies. The resulting evidence would more robustly shed light on the processes contributing to developmental comorbidity.

Several effects were specific to one of the two cohorts, for example, sex differences were evident in NTR, but not in TEDS. For instance, links between externalizing and internalizing overtime were only evident in males. In females on the other hand, changes in externalizing were associated to changes in attention problems, which in turn affected internalizing problems later in life. This might suggest different developmental processes at play in males and females in NTR, compared to TEDS, although differences in the instruments used in the two cohorts might also account for this finding. These results, as well as other cohort‐specific ones, deserve further scrutiny in future work, but are not further discussed herein as they did not replicate across cohorts.

In supplementary analyses we modelled sibling interactions to account for within‐family reciprocal relationships between siblings over time. Overall, we found that accounting for such relationships within‐trait and cross‐trait provided the best fit to the data, suggesting that reciprocal relationships between siblings should be considered as a modest, but detectable, contribution to sibling similarities in developmental models of psychopathology. These longitudinal between‐sibling effects were detected on top of stable and age specific genetic and environmental influences that make children alike within families. All the effects detected were in a positive direction indicating that one twin behaviour reinforced the other twin's behaviour, or the perception of parents thereof. In turn this also indicated lack of evidence for sibling competition or rater contrast effects (Supporting Information).

The approach employed in the current study can be extended to other within‐family designs to help separating relative familial contributions to trait variation. Evidence in this regard can in turn reinforce our confidence in a putative causal relationship. For example, it is intriguing that the effects of conduct/externalizing on emotional problems/internalizing we observed is not only a function of within‐person effects, but it is partly accounted for by between‐sibling relationships. An exciting avenue for future studies is to extend this model by the incorporation of polygenic scores. This may allow extending already existing approaches to estimate gene‐environment interplay and causality in family based designs (Dolan et al., 2019; Minică et al., 2018). In principle implementing the current approach to extended family based designs, may help separating measured direct and indirect genetic effects of siblings, on top of parent‐child relationships.

We should note that the joint consideration of twin and longitudinal data can be applied in many creative ways, for example, to leverage longitudinal sibling‐based designs to infer the development of gene‐environment covariance via phenotypic transmission (De Kort et al., 2014; Dolan et al., 2014). Others have applied longitudinal difference scoring to infer causal relations within a cross‐lagged framework (Moscati et al., 2018; Ritchie et al., 2015). Each of these methods leverages the familial relationships in slightly different ways and highlights the immense value of longitudinal twin and family studies.

It is important to highlight that the RI‐CLPM mitigates confounding by adjusting for unobserved stable covariates with constant effects over measurements (Mund et al., 2021), while the effect of unobserved time‐varying confounders are not accounted for in the model. The constant effect of time‐invariant covariates remains an assumption of the model if these are not directly observed, and modelled accordingly. Alternatively more flexible models could be specified (Mulder & Hamaker, 2021; Mund & Nestler, 2019), but these also require more waves of data. Similarly, the between‐person genetic effects captured by the current model specification are of a stable nature. However, if developmental genetic changes were present (e.g. Pingault et al., 2015) these will likely be pushed into within‐person effects, affecting directed relationships within‐trait and cross‐trait. Including random‐slopes effects in this model might shed light on whether this is the case. However, this model would necessitate of at least four measurement occasions to be specified.

A few other limitations should be highlighted. First, the use of different scales in TEDS and NTR might have influenced our results. For example, although as noted earlier SDQ and CBCL scales have been found to be highly comparable across cohorts (Bartels et al., 2018; Goodman & Scott, 1999; Hendriks et al., 2020; Porsch et al., 2016), differences between constructs might still account for some of the cohort specific effects we observed in our study. On top of these, other between cohort differences could be at play, such as cultural and societal differences. This might be indexed, for example, by differences in estimated twin heritability of the constructs (Supporting Information). A caveat in the interpretation of current findings is that our measures were taken relatively far a part in time (i.e. time‐lags of ∼2 years), although similarly to previous work in this area. As different developmental processes might be at play depending on the timeframe of interest, future investigations on developmental comorbidity should consider different timeframes as a form of triangulation.

Importantly, the RI‐CLM redefines the causal estimand at the within‐person level, focusing on fluctuations around a person stable mean (Hamaker et al., 2015; Lüdtke & Robitzsch, 2021). Hence, it has been suggested that the RI‐CLPM might better capture short‐term changes rather than changes happening over a long period of time (Lüdtke & Robitzsch, 2021; Orth et al., 2021). However, as noted elsewhere (Mulder & Hamaker, 2022), the need of separating within‐person fluctuations and between‐person dynamics is independent from the time‐lags of interest, as there will always be a stable between‐person component that needs to be adjusted for. Nonetheless, one important consideration in our design, is that sibling interactions can be characterized as being amplified through unique environmental processes (De Kort et al., 2012; Dolan et al., 2014). Since non‐shared environmental effects for childhood psychopathology are often transient (e.g. Bartels et al., 2004), we would expect an exponential decay over short time intervals, thus making the relatively long time‐lags employed herein less than ideal. Beyond different time‐intervals, future work could also explore simultaneous (cross‐sectional) sibling effects. Lastly, while we talk about directed influences, these need not be causal influences, there might still be third variable confounding at play, such as time varying covariates not directly accounted for by the model. In this regard triangulation with genetically sensitive approaches, natural experiments, intervention studies, and integration with extended family based designs might help increasing confidence in our findings.

## CONCLUSION

In conclusion, our analyses provided substantive results on trait associations between behavioural problems in two well‐powered longitudinal child cohorts. We found that covariation between psychopathology dimensions is partly attributable to a network of within‐person processes between traits acting on a background of sizeable between‐person differences. Finally extending this approach to family level data we provided a framework that can be extended to other within‐family, genetically sensitive designs in the future.

## AUTHOR CONTRIBUTIONS

Andrea Allegrini: Conceptualization, Formal analysis, Methodology, Software, Visualization, Writing – original draft. Catharina van Beijsterveldt: Writing – review & editing. Dorret Boomsma: Funding acquisition, Writing – review & editing. Kaili Rimfeld: Writing – review & editing. Jean‐Baptiste Pingault: Writing – review & editing. Robert Plomin: Writing – review & editing. Meike Bartels, Conceptualization, Funding acquisition, Project administration, Supervision, Writing – review & editing. Michel Nivard: Conceptualization, Methodology, Project administration, Software, Supervision, Visualization, Writing – original draft.

## CONFLICT OF INTEREST

Kaili Rimfeld serves on the JCPP *Advances* Editorial Advisory Board. The remaining authors have declared that they have no competing or potential conflicts of interest.

## ETHICS STATEMENT

TEDS: Ethical approval for TEDS has been provided by the King's College London Ethics Committee (reference: PNM/09/10‐104). Written parental consent was obtained before data collection. NTR: The study protocols were approved March 16, 2004 by the Central Ethics Committee on Research Involving Human Subjects of the VU University Medical Center, Amsterdam; and May 25, 2017 (NTR‐25‐mei‐2007).

### OPEN RESEARCH BADGES

This article has been awarded Open Materials, Preregistered Research Designs badges. All materials and data are publicly accessible via the Open Science Framework at https://osf.io/dtvc8/. Learn more about the Open Practices badges from the Center for Open Science: https://osf.io/tvyxz/wiki.

## Supporting information

Supporting Information S1

Supporting Information S2

## Data Availability

Researchers can apply for access to the Twins Early Development Study (TEDS) data through their data access mechanism (see www.teds.ac.uk/researchers/teds‐data‐access‐policy). Netherlands Twin Register (NTR) data may be accessed, on approval of the data access committee (email: ntr.datamanagement.fgb@vu.nl).
