# Supplementary material for: Developmental co‐occurrence of psychopathology dimensions in childhood
Source: JCPP Adv. 2022 Sep 5;2(3):e12100. doi: 10.1002/jcv2.12100 (PMC10242955; doi:10.1002/jcv2.12100)
Supplement: Supplementary file 1 — Supporting Information S1 [file JCV2-2-e12100-s002.docx]

Developmental co-occurrence of psychopathology dimensions in childhood

**Author list**

Andrea G Allegrini^1,2^, Toos van Beijsterveldt^3^, Dorret I Boomsma^3,4, 5^, Kaili Rimfeld^1^, Jean-Baptiste Pingault^1,2^, Robert Plomin^1^, Meike Bartels^3, 4^*, Michel G Nivard^3, 4^*

^1^Social, Genetic and Developmental Psychiatry Centre, Institute of Psychiatry, Psychology and Neuroscience, King’s College London, UK

^2^Division of Psychology and Language Sciences, University College London, UK

^3^Department of Biological Psychology, Faculty of behavioral and movement sciences, Vrije Universiteit Amsterdam, Amsterdam, NL

^4^Amsterdam Public Health (APH) Research Institute, Amsterdam University Medical Centre, Amsterdam, NL

^5^ Amsterdam Reproduction and Development (AR&D) Research Institute, Amsterdam University Medical Centre, Amsterdam, NL

**Figure S1**


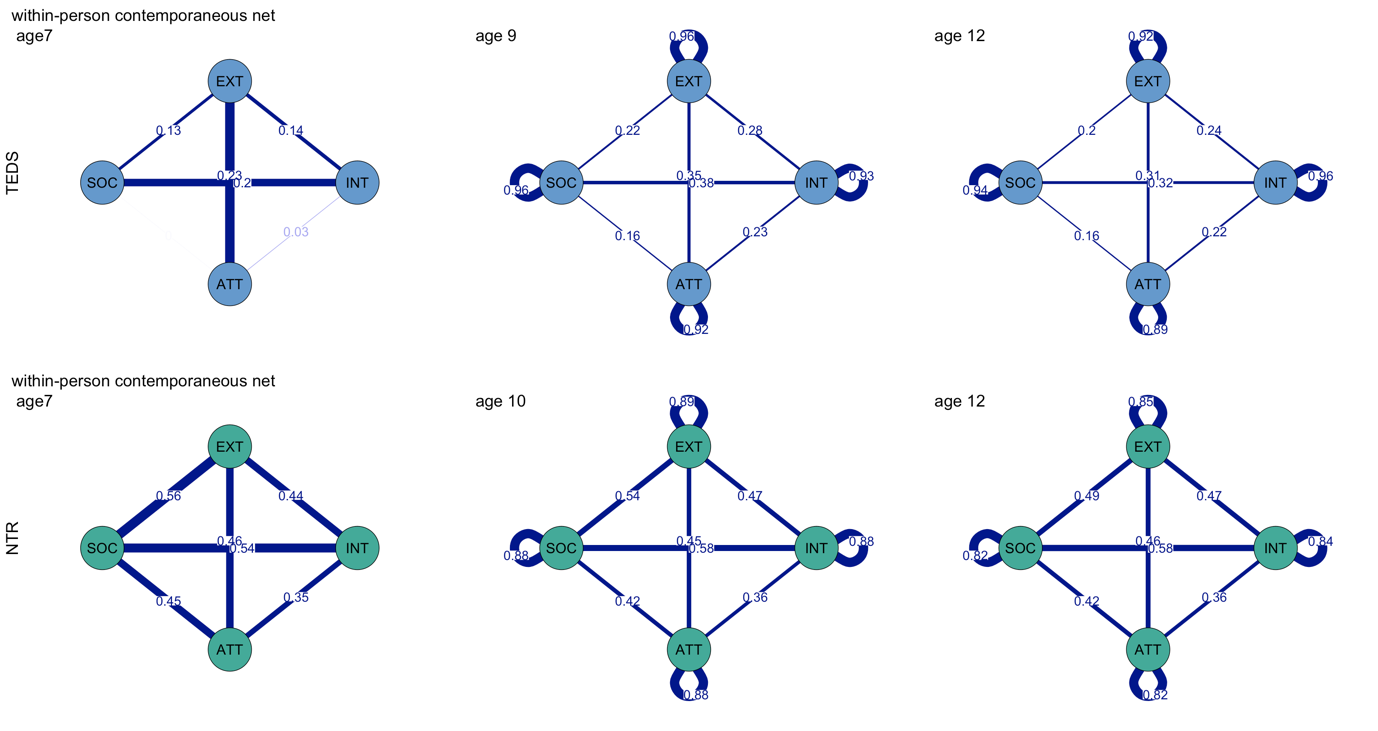


**Figure S1.** Within-person contemporaneous net. Edges width and labels indicate and quantify the strength of relationships between nodes. For every measurement occasion (7 to 12) edges represent simultaneous within person correlations cross-trait, and residual variance (node covariance). **Note.** SDQ/CBCL acronyms: EXT = conduct/externalizing, ATT = hyperactivity-inattention/inattention, INT = emotional problems/internalizing, SOC = peer problems/social problems. **Note.** SDQ/CBCL acronyms: EXT = conduct/externalizing, ATT = hyperactivity-inattention/inattention, INT = emotional problems/internalizing, SOC = peer problems/social problems. Blue edges survived FDR correction, grey edges nominal significance (alpha <.05).

**Figure S2**


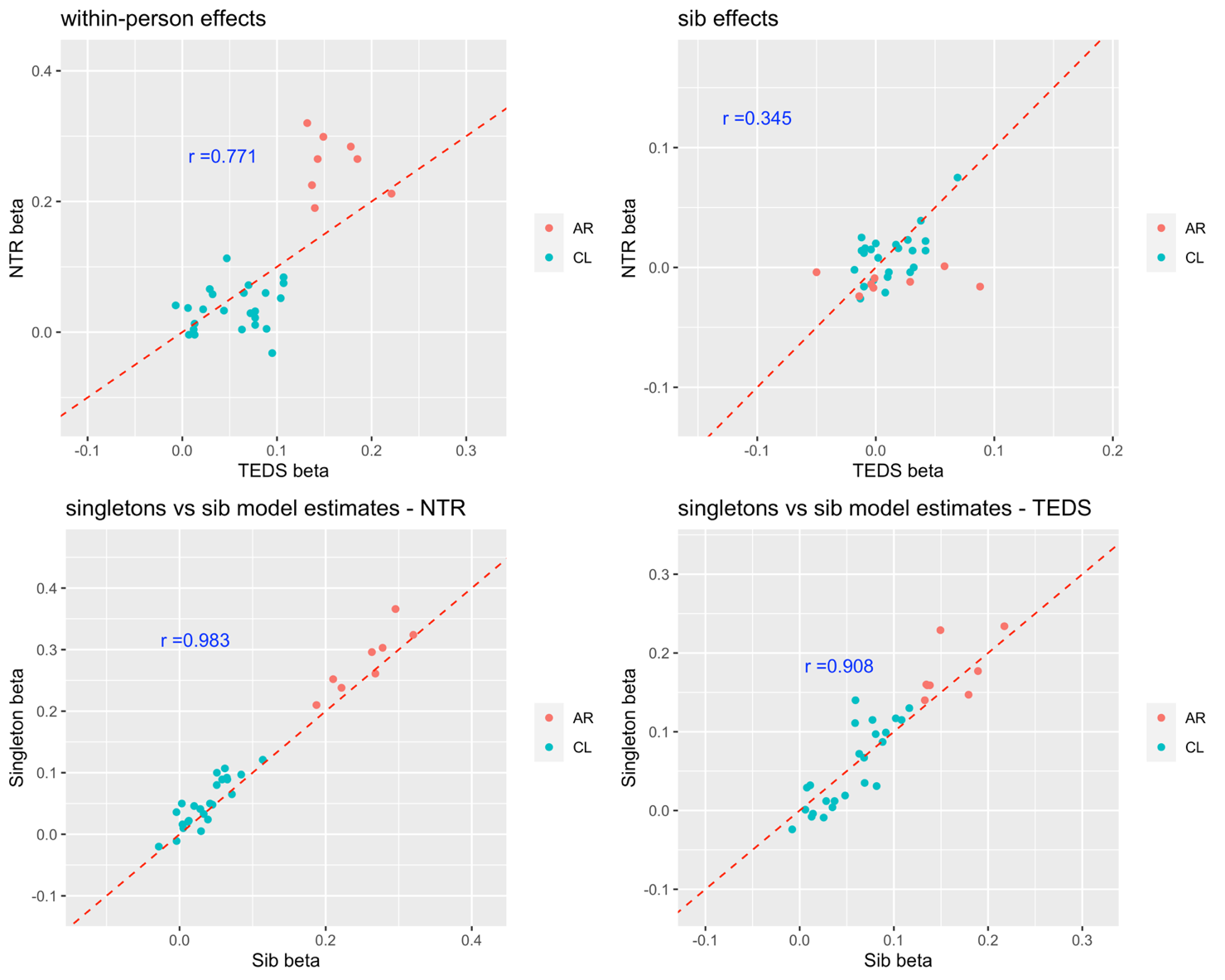


**Figure S2.** Comparison of within-person effect sizes estimated in the RI-CLPM vs the wf-RI-CLPM. Left panel comparison between models in NTR. Right panel: comparison in TEDS. AR = autoregressive (carry-over) effects. CL = cross-lagged effects. Dashed line: x=y.

**Figure S3**


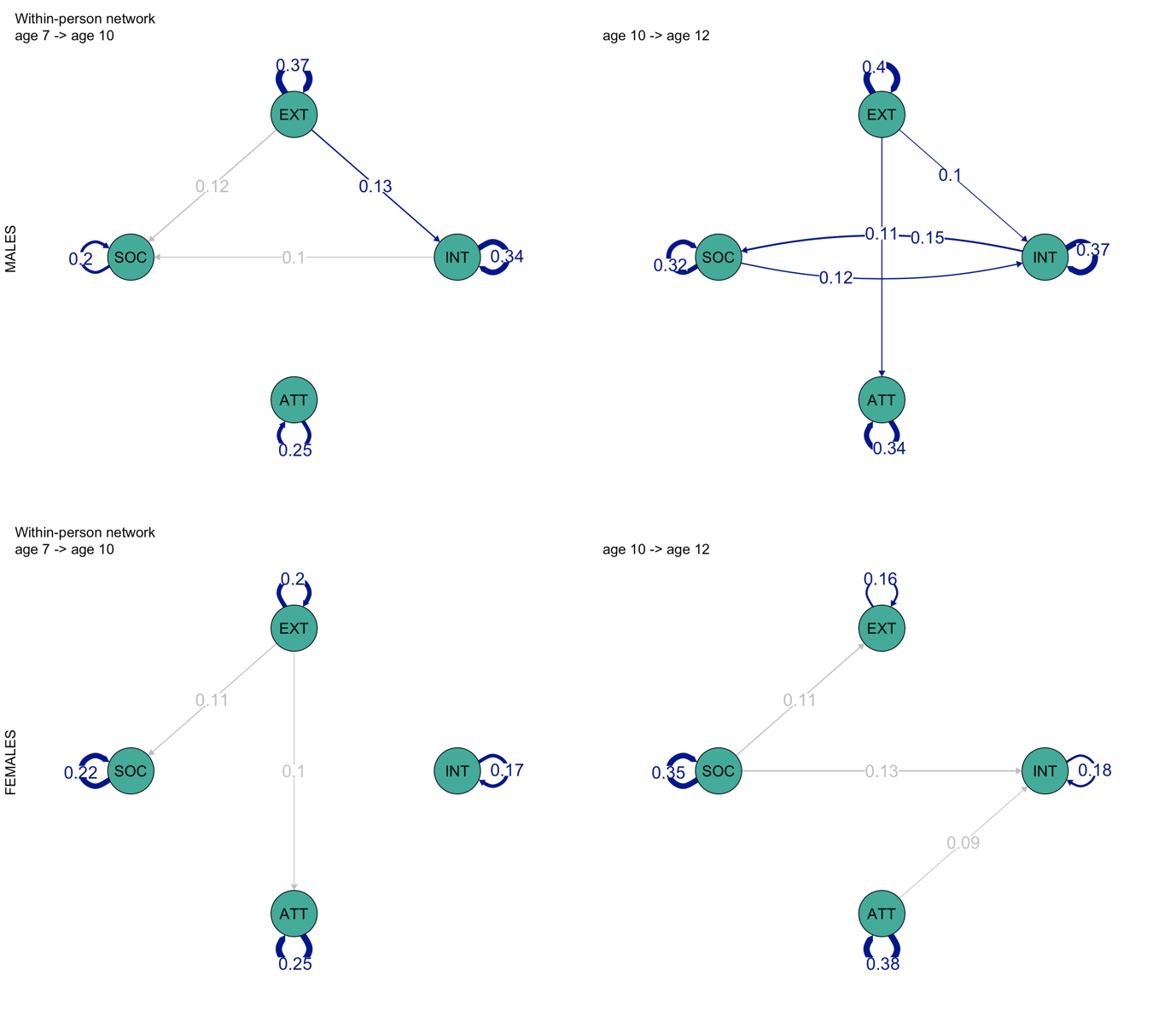


**Figure S3.** Between-person and within-person (directed) networks of relationships in Males (top) and Females (bottom) obtained from the RI-CLPM in NTR. Nodes represent the measure of interest (the random intercept in the case of between-person networks, and residual deviation of measurement occasion for the within-person network). Edges width and labels indicate and quantify the strength of relationships between nodes, and in the case of within-person networks also the temporal direction of the effect. For every time lag (7-10 and 10->12) edges represent directional effects within-trait (self-pointing arrow) or cross-trait. **Note.** Acronyms: EXT = conduct/externalizing, ATT = hyperactivity/ hyperactivity-inattention, INT = emotional problems/internalizing, SOC = peer problems/social problems. Blue edges survived FDR correction, grey edges nominal significance (alpha <.05).

**Figure S4**


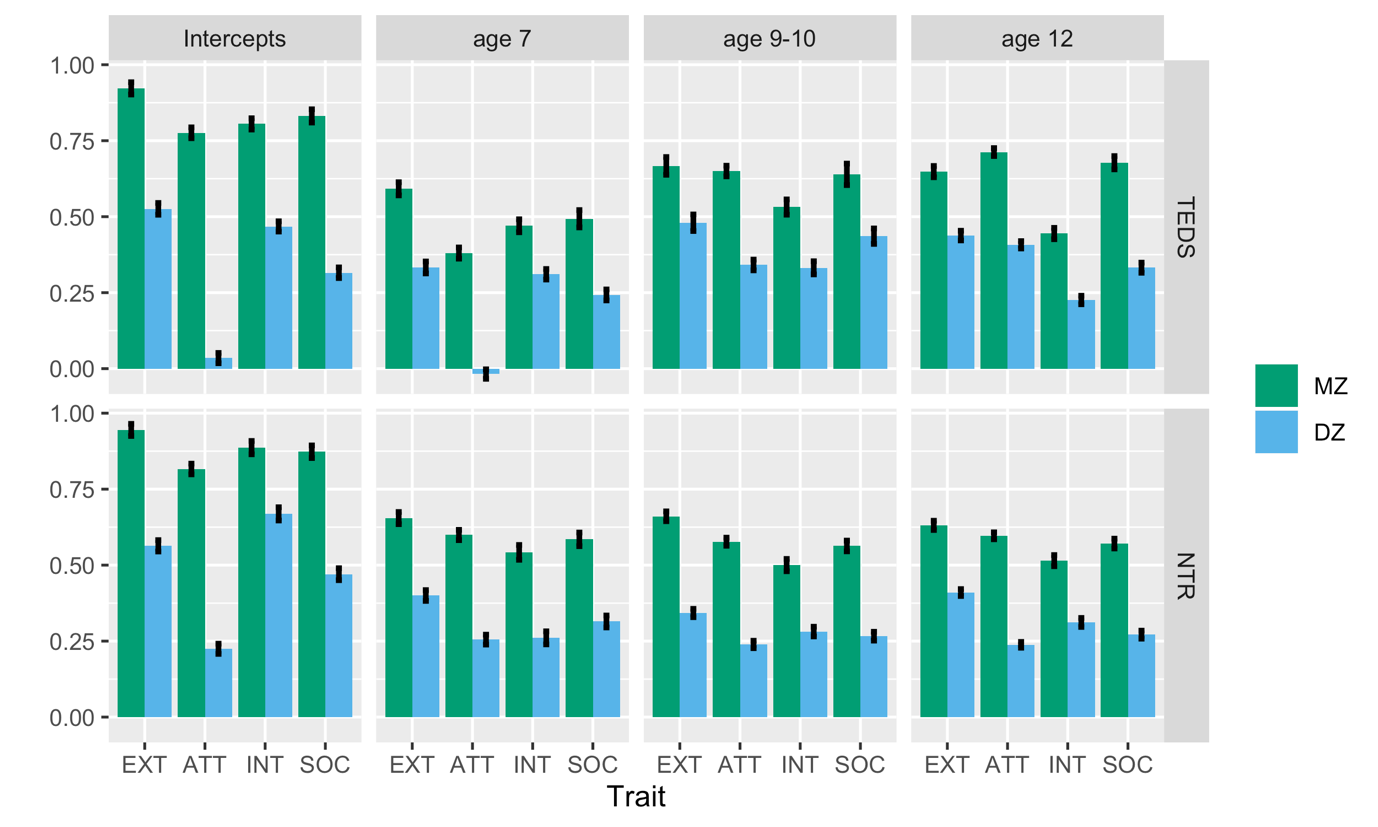


**Figure S4** Twin correlations for random intercepts and residual deviations in the wf-RI-CLPM in TEDS and NTR.

**Figure S5**


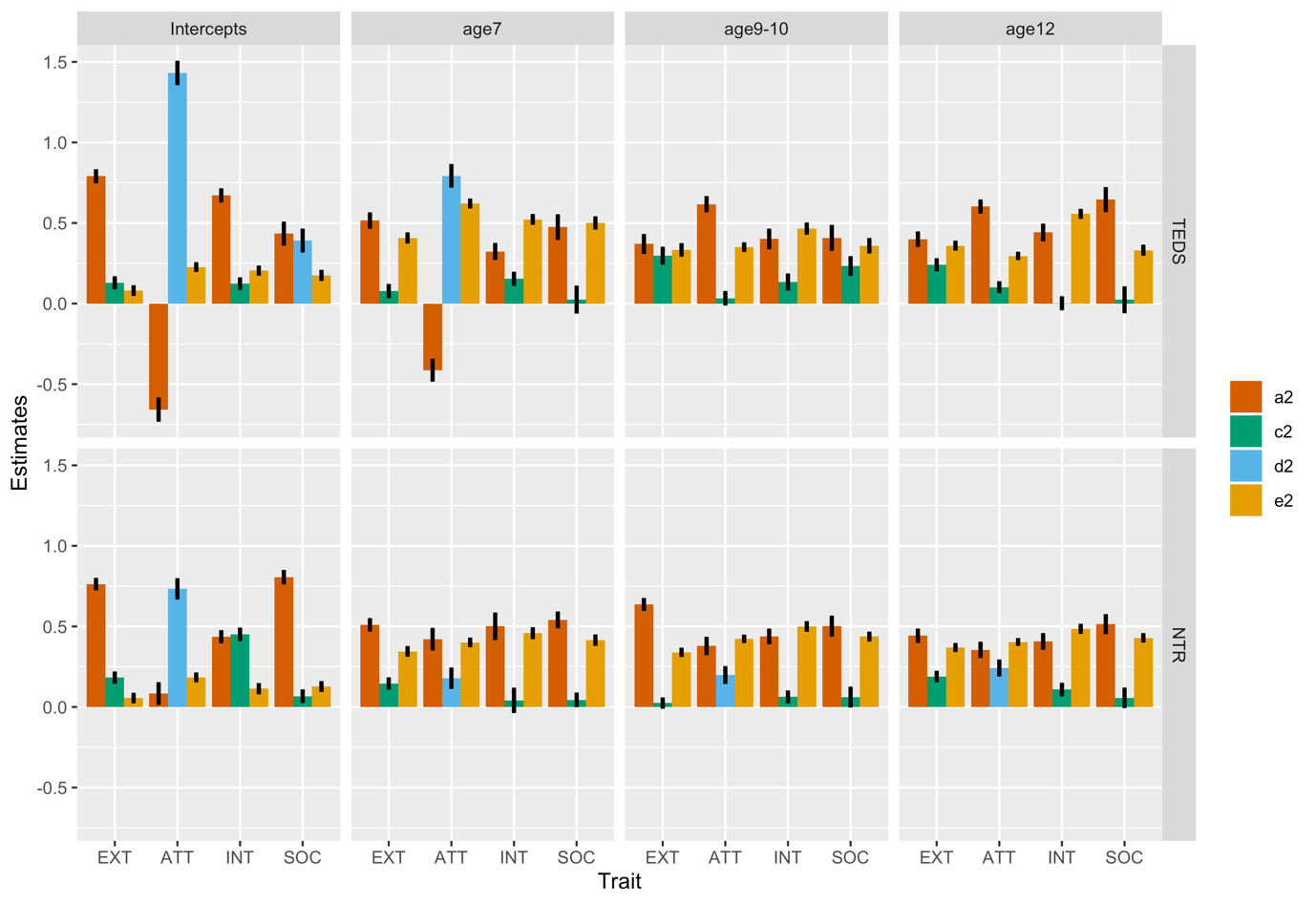


**Figure S5.** Variance components for random intercepts and age specific residual variances estimated from MZ and DZ correlations, corresponding to shared additive (a^2^), dominance (d^2^), environmental effects (c^2^), and unique environmental effects (e^2^). Note the over-dominance for attention problems (ATT) is consistent with DZ correlations < .25* the MZ correlation.

**Supporting Information 1**

**Sibling interactions in the context of this study**

Sibling interactions can result in cooperation and contrast effects, which respectively index the extent to which siblings will tend to imitate/stimulate each other increasing their phenotypic similarity, or contrast each other, decreasing their resemblance (Carey, 1986). Siblings interactions have been investigated in the quantitative genetics literature especially in regard to externalizing traits such as hyperactivity and conduct disorder (Thapar et al., 1995, Simonoff et al., 1998, Rebollo and Boomsma, 2006, Boomsma, 2014). A typical observation for traits such as these is the low dizygotic twin correlations compared to monozygotic twin correlations, which can be attributed to genetic dominance (interactions of two alleles at the same locus) or contrast effects. Results of such studies are mixed, finding that contrast effects are either due to siblings interactions (Thapar et al., 1995) or parental bias (Simonoff et al., 1998), where the difference in, for example, hyperactivity ratings within sibling pairs index parental perception rather than a true difference between siblings. Importantly contrast effects can mask dominance (Simonoff et al., 1998). This picture is complicated by issues of power, which makes it difficult to estimate contrast versus dominant effects concurrently (Rebollo and Boomsma, 2006). With some exceptions (Carey, 1986, Dolan et al., 2014, Rietveld et al., 2003, Rietveld et al., 2004) these studies infer contrast effects rather than directly quantifying the reciprocal effects of sibling phenotypes within families. Here we investigate within-family reciprocal directed influences between siblings across several behavioural problems over time.

Sibling interactions can be separated into cooperative and contrast effects, indicated in our models by positive or negative predictions of one twin behaviour at one time point on the other twin behaviour at another time point. Within age specific residuals, however, sibling interactions would load on either d^2^ (dominance effects) or c^2^ (shared environmental factors), depending on whether these are contrast or cooperative effects respectively. There is evidence, at least for childhood hyperactivity, that maternal ratings may suffer from rater contrast (Bartels et al., 2007, Simonoff et al., 1998), as such contrast and cooperative effects between siblings are rather an index of parental perception of siblings interacting rather than an index of true behavioural interaction between siblings. Again, these effects will inflate d^2^ and c^2^ estimates, decreasing or increasing DZ similarities respectively. Contrast effects can also mimic dominance (Simonoff et al., 1998) and this would partly explain the out of bounds estimates we find in TEDS for the latent time invariant hyperactivity trait. We observe overdominance (d^2^ above 1) in TEDS for hyperactivity which is likely a combination of true dominance effects (that we also observe in NTR for this trait) and competition effects (which might in part be inflated by parent bias).

We did not attempt to formally distinguish between contrast and cooperative effects when separating genetic and environmental influences at each age. However, no dominance effects were observed for internalizing problems or externalizing problems, which were the two problem behaviours involved in the between sibling effects that replicated across cohorts. In this regard, if rater contrast accounted for the underlying pattern of relationships, we should have observed negative directed relationships between siblings. That is, the more one twin is rated high on a construct the less the other twin will be perceived to be problematic and thus rated accordingly. However, this effect is in the opposite direction than what we generally observed, with higher ratings on one trait corresponding to higher ratings on the same or other traits. We could have the reverse where globally we perceive siblings to have same levels of problem behaviours, this would inflate c^2^ estimates as observed elsewhere (Allegrini et al., 2019). However, we do not observe consistent c^2^ estimates for any of the replicated findings across cohorts, both within and between sibling effects.

**Supporting Information 2**

**Genetic and environmental influences in the context of the wfRI-CLPM**

Every twin phenotypic value at a given time point is a function of the person genetic contributions at that time (*h*^2^, additive or dominance effects), shared environmental (*c^2^*) and unique environmental (plus error; *e^2^*) variance. In the wfRI-CLPM we further separate genetic and environmental effects (*h^2^ + c^2^ + e^2^)* of the stable between-person component, and time specific (*h^2^_t_ + c^2^_t_ + e^2^_t_)* within person *residuals*, which can be parsed out into genetic and environmental component of variance on top of directed within-person effects from one time point to the next (independent of between person time invariant effects and time specific residual cross-twin cross-trait covariance):

For phenotype ‘p’ of person *i* at time point *t*:

*p_it =_ h^2^_t_ + c^2^_t_ + e^2^_t_ +* $\alpha$*_t_p_i,t-1_ +* $\beta$*_t_q_i,t-1_ +* $\alpha$*_t_p_ji,t-1_ +* $\beta$*_t_q_ji,t-1_*

that is, the phenotypic value of each twin at a certain time point is given by the within person effects and the sibling effects from the previous time point (within and cross trait), and an age specific residual that is separated in shared genetic, shared environmental, and unique environmental influences, after controlling for between person stable effects overtime. Modelling the between person covariances separately for MZ and DZ twins as a multi-group analysis, we can thus estimate genetic and environmental relative contributions to the variation in latent stable traits (the random intercepts) and age specific effects (the time specific residuals for each trait). Based on Falconer’s formula, we can estimate genetic (*h^2^* and *d^2^*) and environmental (*c^2^* and *e^2^*) contributions by comparing MZ twins correlations (rMZ) to DZ twins correlations (rDZ) as follows:

h^2^ = 2*(r_MZ_ - r_DZ_)

c^2^ = 2*r_DZ_ – r_MZ_

For traits where non additive effects are evident (negative c^2^ represented by r_MZ_ being more than twice r_DZ_) we estimate additive vs dominance effects as follows:

a^2^ = 4*r_DZ_ – r_MZ_

d^2^ = 2*r_MZ_ – 4*r_DZ_

Unique environmental contributions (plus error) can be estimated as follows:

e^2^ = 1= r_MZ_

An ADE or ACE model can be specified a priori upon inspection of the covariance structure for each trait.

**References**

ALLEGRINI, A. G., CHEESMAN, R., RIMFELD, K., SELZAM, S., PINGAULT, J.-B., ELEY, T. & PLOMIN, R. (2019). The p factor: Genetic analyses support a general dimension of psychopathology in childhood and adolescence. *bioRxiv***,** 591354.

BARTELS, M., BOOMSMA, D. I., HUDZIAK, J. J., VAN BEIJSTERVELDT, T. C. & VAN DEN OORD, E. J. (2007). Twins and the study of rater (dis) agreement. *Psychological methods,* 12**,** 451.

BOOMSMA, D. I. (2014). Sibling interaction effects. *Wiley StatsRef: Statistics Reference Online***,** 1-3.

CAREY, G. (1986). Sibling imitation and contrast effects. *Behavior Genetics,* 16**,** 319-341.

DOLAN, C. V., DE KORT, J. M., VAN BEIJSTERVELDT, T. C., BARTELS, M. & BOOMSMA, D. I. (2014). GE covariance through phenotype to environment transmission: an assessment in longitudinal twin data and application to childhood anxiety. *Behavior Genetics,* 44**,** 240-253.

REBOLLO, I. & BOOMSMA, D. I. (2006). Genetic analysis of anger: genetic dominance or competitive sibling interaction. *Behavior Genetics,* 36**,** 216-228.

RIETVELD, M., POSTHUMA, D., DOLAN, C. & BOOMSMA, D. (2003). ADHD: sibling interaction or dominance: an evaluation of statistical power. *Behavior Genetics,* 33**,** 247-255.

RIETVELD, M. J., HUDZIAK, J. J., BARTELS, M., VAN BEIJSTERVELDT, C. & BOOMSMA, D. I. (2004). Heritability of attention problems in children: longitudinal results from a study of twins, age 3 to 12. *Journal of Child Psychology and Psychiatry,* 45**,** 577-588.

SIMONOFF, E., PICKLES, A., HERVAS, A., SILBERG, J., RUTTER, M. & EAVES, L. (1998). Genetic influences on childhood hyperactivity: Contrast effects imply parental rating bias, not sibling interaction. *Psychological Medicine,* 28**,** 825-837.

THAPAR, A., HERVAS, A. & MCGUFFIN, P. (1995). Childhood hyperactivity scores are highly heritable and show sibling competition effects: Twin study evidence. *Behavior Genetics,* 25**,** 537-544.
